# Gender and race in neurotrauma: part 2–underrepresentation in clinical trial enrollment and impact on clinical outcomes

**DOI:** 10.3389/fneur.2025.1587632

**Published:** 2025-05-13

**Authors:** Tea C. Sue, Isabella F. Churchill, Ann M. Parr, Eve C. Tsai

**Affiliations:** ^1^Faculty of Medicine, University of Ottawa, Ottawa, ON, Canada; ^2^Department of Neurosurgery, University of Minnesota, Minneapolis, MN, United States; ^3^Department of Surgery, Faculty of Medicine, University of Ottawa, Ottawa, ON, Canada; ^4^Neuroscience Program, Ottawa Hospital Research Institute, Ottawa, ON, Canada

**Keywords:** neurotrauma, gender, race, intersectionality, trial representation

## Abstract

The underrepresentation of women and racial minorities in clinical trials populations remains a persistent challenge across many medical specialties, including Neurosurgery. A diverse research cohort brings varied perspectives and experiences, which can lead to more innovative solutions to medical problems, generalizable findings, and the foundations to provide culturally competent care to the populations most affected by the condition at hand. The importance of representative Neurotrauma trial populations cannot be overstated, as results are essential to inform decision making and gender and race have both been shown to significantly influence patient outcomes, as seen in the traumatic brain injury and spinal cord injury populations. Although the path towards gender and racial parity in clinical trial participants has been slow, numerous actions have been taken, including the FDA Safety and Innovation Act (2012) and Omnibus Reform Act (2022) on a systemic level. In this paper, we aimed to explore the barriers to and implications of inadequate representation in neurotrauma trials to outline a roadmap towards more diverse trial inclusion and retention. Key strategies moving forward include recruiting a diverse research team, developing flexible study protocols that support the varying needs of individuals of different backgrounds, establishing methods of data analysis that control for social and demographic factors instead of excluding individuals from participating, introducing patient navigators, reflecting on systemically engrained biases, implementing mandatory reporting of gender and race data, establishing and analyzing policies that keep researchers accountable towards goals of inclusive recruitment, and identifying and addressing unique barriers that individuals at the intersection of gender and racial minority status face.

## Introduction

The persistent underrepresentation of women and racial minorities in clinical trials remains a significant challenge across various medical fields (1–3). Despite the increasing incidence of diseases and conditions among these groups, inclusion in clinical research has not increased concomitantly, leading to a critical gap in research representation (4, 5). This underrepresentation not only limits the generalizability of research findings but also contributes to suboptimal healthcare outcomes for these populations, partially attributed to varying pharmacodynamics and pharmacokinetics across different gender and racial groups (6–9). Numerous studies have documented lower enrollment rates for minority groups compared to white participants in clinical trials (4, 5, 8). Furthermore, government-funded clinical trials have demonstrated higher gender and racial disparities compared to industry-funded trials, underscoring the need for increased focus on diverse enrollment in publicly funded research (4, 5). This article expands on a prior perspective piece that addressed the underrepresentation and challenges faced by women and racial minorities in neurosurgery, with an emphasis on strategies to work towards enhancing workforce diversity (10).

Representation within clinical trials is critical given the differing effects of various devices or medications based on such demographic differences (6, 9). Minority groups often experience different health outcomes and responses to treatment (5–7). Including these groups in research helps identify and address health disparities, leading to more equitable healthcare (5–7). For instance, women and people of color are more likely to sustain a violent traumatic brain injury (TBI) but are less likely to seek care and receive aggressive treatment, highlighting the need for inclusive research (11). Most concretely, evidence from clinical trials is utilized by the FDA to approve medical and surgical devices in the USA, and therefore, representation within these trial populations in comparison to the epidemiology of the disease the devices are utilized for, is of utmost importance (1). In an effort to address the existing disparities, the FDA Safety and Innovation Act was passed in 2012, mandating the publishing of gender distribution and encouraging voluntary reporting of race/ethnic data of trial participants (3).

Diverse research participation in Neurosurgery is crucial for several reasons, as highlighted by the current literature. First, diversity in research serves as a catalyst for innovation and reduces bias, ultimately improving patient care (12–14). Clinical trials that adequately represent the diverse population affected by the selected disease ensures that research outcomes are applicable to a broader population, which is essential for developing effective treatments and interventions for all demographic groups (1, 3). A study by Siddiqui et al. (1) investigated gender and racial diversity amongst 33 neurosurgical device trials and found that both before and after the introduction of the FDA Safety and Innovation Act, females and minorities were underrepresented in clinical trials, emphasizing the persistence of this disparity (1). This trial also highlighted that for clinical trial populations where men (carotid stenosis) or females (aneurysms) are predominantly affected, the trial population reflects these proportions, however, when these same diseases predominantly affect a minority group, this is not similarly reflected, indicating a particular need to improve racial representation in line with the epidemiology of the specific disease (1). To build upon this, the FDA released their Omnibus Reform Act in 2022 with new mandates outlining the recruitment and retention of diverse trial participants, requiring submission of diversity action plans that are adaptable to meet diverse enrollment goals.

Diverse research participation helps in understanding how social determinants of health interplay with physical illness, which is crucial for providing culturally competent care. A diverse workforce that can personally provide these insights can improve clinical outcomes in neurosurgical patients by ensuring that care strategies are tailored to the specific needs of these populations (15). Unfortunately, the lack of diversity in the neurosurgical workforce has a downstream effect on mentorship and representation (14, 16). Minority medical students often lack mentors who share their background, which may hinder recruitment into neurosurgical training programs. Programs that actively promote diversity can help create a more inclusive and supportive environment, encouraging more minority students to pursue careers in Neurosurgery (14, 16). Achieving diversity requires systemic changes, such as linking national program rankings and accreditation to the hiring and retention of minority residents and faculty. This can help create a more equitable and inclusive neurosurgical workforce, which is essential for retaining talent and improving patient care.

There are extensive barriers to clinical trial participation including financial barriers, logistical concerns, and stringent inclusion criteria which may inadvertently exclude patients with certain social barriers or those from ethnic groups with a higher proportion of excluded comorbidities (2, 8). Furthermore, historical mistreatment and socioeconomic barriers contribute to the reluctance of minority groups to participate in clinical trials. Efforts to build trust, reduce trial burdens, and provide equitable access to research opportunities are necessary to overcome these barriers and ensure diverse participation (15, 17). By addressing these factors, the neurosurgical field can advance toward more inclusive and equitable research practices, ultimately enhancing patient care and outcomes across diverse populations. As such, the aim of this paper was to investigate the barriers to and implications of unrepresentative neurosurgical trial participation to inform a roadmap towards more diverse trial inclusion with the hope of improving the applicability of research findings to the population at large.

### Decreased reporting in neurotrauma trials

The importance of clinical trial results in establishing the safety and efficacy of new medical interventions cannot be overstated, and the evaluation of these outcomes is essential for informed medical decision-making. However, the effectiveness and safety of interventions may vary across different population subgroups. Notably, racial minorities within the neurosurgery/neurotrauma literature remain underrepresented, posing challenges to the generalizability of findings.

Racial and ethnic minorities, particularly black participants, are underrepresented in traumatic brain injury (TBI) clinical trials (15). About 25% of the analyzed trials reported a racial diversity index below 1, indicating substantial racial disparity. Notably, industry-funded trials exhibited a 26% higher likelihood of racial disparities compared to federally funded trials. Indeed, most scientific studies (78%) do not even report racial/ethnic demographic information (15).

In the context of spine surgery outcomes, demographic factors, including race, can significantly influence patient outcomes. A systematic review of randomized controlled clinical trials published in high-impact spine journals between January 2012 and 2022 revealed a low frequency of reporting demographic information. Only a small percentage of studies reported race, and the reporting frequency did not vary based on the publishing journal (18). The study emphasizes the importance of increased reporting of demographic information in spine surgery research to enhance the external validity and generalizability of findings, particularly considering the impact of demographic factors on patient outcomes and disparities in care.

With respect to the effect of gender on patient recruitment, the gender of authors has been correlated with proportion of female participant recruitment. Neurosurgery clinical trials with both first and senior authors being female exhibited higher female enrollment compared to trials with male authorship (19, 20). This correlation persisted across various subset analyses, including funding source, phase, randomization, drug/device trials, and geographic location.

Most epidemiologic traumatic brain injury (TBI) studies report that men are more affected than women, partially explained by the increased likelihood of men to be involved in physical altercations or contact sports that may predispose them to head trauma (21). However, if head trauma secondary to intimate partner violence were to be reported within this population, it is estimated that greater than 31 million women would be included – a statistic that would significantly influence the currently reported demographics of the TBI population (22). Therefore, TBI incidence in women may be equal or even greater than that in men (23, 24). Nonetheless, women are markedly underrepresented in TBI clinical trial enrollment (15). Approximately 50% of the analyzed trials exhibited significant gender disparity, with a gender ratio of less than 0.4. This issue is more pronounced in federally funded TBI trials, which demonstrate greater gender disparity rates compared to those funded by industry sources. These findings highlight the persistent underrepresentation of both women and racial/ethnic minorities in TBI clinical trials, underscoring the need for targeted efforts to enhance diversity and ensure equitable representation in neurotrauma research (21).

### Differences in neurotrauma outcomes

There are mixed results for poorer outcomes based on gender following neurotrauma injuries. In a study involving 7145 patients with acute traumatic brain injury (TBI) it was found that mortality rates and unfavorable outcomes were comparable between males (7.48 and 16.05%, respectively) and females (7.22 and 17.23%, respectively) with acute TBI (25). When looking at biomarkers, evaluation of GFAP and UCH-L1 biomarkers in a cohort of trauma patients showed no significant gender-related differences in diagnostic accuracy for mild TBI or traumatic intracranial lesions (26). While patterns of biomarker elevation were similar, male patients had significantly higher UCH-L1 concentrations within 24 h of injury. Similarly, a study on acute traumatic cervical spinal cord injuries (SCI) found comparable outcomes in therapeutic approaches, length-of-stay, mortality, and discharge disposition between genders (27). However, while both genders exhibited similar rates of post-SCI complications, women showed a trend for higher psychiatric complications and deep venous thrombosis. Regarding managing neurological emergencies, significant gender differences were found in symptom onset time, hospital transportation, neuroimaging, admission rates, length of stay, or disposition. However, females were more likely to present with headache, and a higher proportion had health insurance coverage compared to males. In contrast, another study analyzing SCI data (546 patients) revealed differences in the mechanisms and types of injuries between genders (28). Women with SCI were more susceptible to falls and suicide attempts, while men were more involved in motorcycle accidents and falls from height. Women involved in motor vehicle crashes showed more significant lumbar spine lesions, whereas men developed mainly cervical spine injury (28).

Although poorer outcomes for racial minorities are clear, neurotrauma is the least investigated sub-specialty for disparities in neurosurgery (29). African Americans (AA) with mild TBI reported greater headache pain, pain catastrophizing, higher pain sensitivity, and worse pain modulation compared to Caucasians (30). Review of TBI literature identified significant racial/ethnic disparities in TBI outcomes, with American Indian/Alaska Natives patients having the highest TBI-related death rates. Black patients were more likely to incur TBI from violence, and minorities had worse functional outcomes compared to Non-Hispanic Caucasian (31). Analysis of the National Trauma Data Bank (NTDB) revealed racial disparities in SCI outcomes, including differences in length of hospital stay, complications, and patient disposition. African Americans and Native Americans had longer hospital stays and higher rates of complications, while African Americans and Asians were less likely to be discharged to acute rehabilitation programs (32). A study using the National Sample Program of the NTDB explored predictors of morbidity and mortality after spinal trauma, highlighting the impact of race/ethnicity and insurance status on outcomes (33). Non-Caucasian and African American race increased the risk of mortality, and lack of insurance increased mortality while decreasing hospital days, ICU days, and ventilator time. In regard to spine surgery, one study revealed that African Americans had higher odds of in-hospital complications and mortality compared to Caucasians following cervical spine surgery (34). Further, analysis of the NTDB from 2017 to 2019 demonstrated racial disparities in time to surgical decompression for central cord syndrome (35). Black patients, female patients, and those treated at community hospitals were less likely to receive early surgery for central cord syndrome, emphasizing demographic disparities in timely intervention.

## Discussion

Although investments in research have led to significant clinical advancements, the underrepresented population may not benefit from such discoveries, given inadequate representation in the corresponding clinical studies (36). As identified in a comprehensive review published in 2022, progress has been made in representing Caucasian women in clinical trials, however, steps towards including racial minorities have been limited, further compounding the health disparities faced by these groups (36). Although women and racial minorities are more likely to sustain a violent TBI, most associated studies are conducted by Caucasian men and Caucasian male participants are disproportionately enrolled, consequently affecting the applicability of the results to the population most affected by violent TBIs (11, 37). The underrepresentation of women and minorities in TBI trials has shown no improvement from 2008 to 2022 (15). Trials with greater racial diversity tend to have lower completion and retention rates, especially for Black participants, which may be influenced by factors such as lack of diversity in research teams, trust issues, and cultural differences. Although racial minorities are approximately twice as likely to die of a TBI in comparison to their male counterparts, ongoing research efforts are not addressing this disparity (11). Furthermore, there is a lack of robust data on the intersectional effects of race/ethnicity and gender on TBI outcomes and recovery trajectories. Addressing these disparities is crucial for ensuring equitable access to effective TBI treatments and avoiding biased evidence that fails to represent all populations. Targeted efforts to increase diversity, thorough analyses of gender and race differences, and consideration of sociocultural factors are essential for improving TBI research and clinical practices.

Improving the representation amongst the clinical trial pool of participants will encourage the adequate representation in associated studies, hopefully, eliciting both the differing and overlapping needs of various populations. Unfortunately, this is further impacted by the underreporting of race data in clinical trials (19). With respect to clinical practice, the United States Food and Drug Administration (FDA) uses evidence from clinical trials to determine both safety and utility, however, extrapolation of this data has always been limited given the unrepresentative study populations in comparison to the population at large. To address this gap, the FDA Safety and Innovation Act was enacted to mandate reporting of specific study demographics in new neurosurgical device applications, however, it has yet to have an impact on the composition of the corresponding clinical trial populations (1). Therefore, not only are efforts to improve gender and racial diversity within clinical trial populations required, but stricter and heavy enforcement of such measures may be required to yield a significant impact.

### Reasons for low reporting

Despite the increase in the awareness of healthcare disparities faced by minorities and underrepresented groups, there is still a paucity of race reporting in surgical trials, which represents substantive concerns and has been scrutinized in the literature, especially in international trials. Potential explanations for this may be fear or mistrust from these groups, inappropriate exclusion criteria, poorly designed trials, barriers to access, and consent issues (38). Additionally, in trials with qualitative methodologies or characterized by modest sample sizes, the deliberate exclusion of racial categories may be observed as a precautionary measure to forestall potential participant identification. Barriers for underrepresented minorities entering clinical trials may include socioeconomic and health literacy issues (39). Further, clinical trial burden such as length of the visit and increased visits needed might make minority participants less likely to participate (40). When a lack of reporting is present, authors should acknowledge this limitation, yet the literature shows that they rarely include this in their methodological limitations or articulate a rationale for the exclusion of race.

### Roadmap to closing the gender and racial gap in neurotrauma research participation

Although the road to improving gender and racial disparities in neurotrauma research may not be linear, we have outlined a number of efforts that can be implemented to advance this goal.

To recruit a diverse trial population, it is essential to foster a diverse research team. This involves not only enhancing departmental diversity, but also implementing active strategies for mentoring, training, and retaining gender and racial minorities interested in Neurosurgery (15, 41). Establishing a diverse research team, specifically with individuals from the predominantly affected population, may enhance community engagement and provide an educated and personally driven perspective on the topic at hand. With a diverse team comes a heterogeneous set of skills and language proficiencies which may help facilitate translation efforts and consequently reduce the number of females or minority individuals who are eager to participate but unfortunately excluded due to English proficiency (11).

Although willingness to be included cannot be ignored as a contributing factor to the predominant enrollment of Caucasian males in clinical trials, it is important to consider the time constraints, resource needs and cultural backgrounds of all participants during protocol design and patient recruitment (15). Offering tailored support for obstacles that may disproportionately affect women and minorities from participating, including provision of taxi chits, childcare services or religious accommodations, may help minimize the barriers that quickly prevent females, racialized minorities, or those from different socioeconomic statuses from participation in these trials (15). Additionally, instead of applying strict inclusion criteria that may preemptively exclude individuals of demographic backgrounds who naturally have higher rates of comorbid conditions, employing tailored methods of data analysis to later account for these factors may provide more inclusive results transferrable to the affected population (8). To augment this, implementation of patient navigators – individuals who identify barriers and develop strategies to overcome them while often being demographically concordant and multilingual – have proven effective in improving participant recruitment, enrollment, and retention in oncology clinical trials (2). However, system-level change takes time, and despite several proposed solutions, the practicality and feasibility of implementing these resources and additional supports limits the possibility of seeing rapid changes.

Given the federal government’s uniquely powerful position as the funder and regulator of many ongoing research projects, it has a crucial role to play in promoting the diversity of the workforce and included study populations (5). As was discussed above regarding the evaluation of the FDA Safety and Innovation Act, promoting and analyzing the implementation of inclusive policies is crucial to understand the utility of such steps (5, 36). Furthermore, establishing policies that require inclusive recruitment to be both planned and actively implemented, in addition to policies that terminate projects that circumvent this, are required to ensure improvements in attaining representative study populations (15, 36).

As highlighted above, there are many ways we could take a step forward towards improving representation within departments, amongst trainees, on research teams, and in clinical trial populations, however, it is also important to take a step back and objectively reflect on systemically engrained biases that contribute to the current state of diversity. Engrained biases may affect how patients are treated by healthcare professionals, the speed at which patients are seen, and what is focused on during an exam; ultimately influencing the overall perception of the patient and exam findings - factors which may influence their compatibility with trial enrollment. Inclusion of a diverse research team, developing community partnerships, and improving communication strategies to build trust and a tailored understanding, may help mitigate such bias.

The importance of intersectionality—considering the interplay between gender, race, and other social determinants of health—is increasingly recognized in neurotrauma research (42). Although ongoing initiatives aim to increase diversity in clinical trials, progress has been slow, and challenges remain (43). By addressing these issues and implementing targeted strategies, neurotrauma research can work towards closing the gender and racial gap in research participation. This is an essential next step for the neurosurgical community in order to foster more inclusive and equitable health outcomes, ensuring that research findings are more reflective of and applicable to the diverse populations affected by neurotrauma.

## Data Availability

The original contributions presented in the study are included in the article/supplementary material, further inquiries can be directed to the corresponding author.
